# Antegrade Concorde position for leveraging the lateral supracerebellar infratentorial approach

**DOI:** 10.1016/j.bas.2026.106280

**Published:** 2026-08-07

**Authors:** Peter Truckenmueller, Nadja Katharina Moser, Bianca Bossi, Lucius Samo Fekonja, Anton Früh, Lars Wessels, Kiarash Ferdowssian, Julia Sophie Onken, Peter Vajkoczy

**Affiliations:** a Department of Neurosurgery, Charité - University Hospital, Berlin, Germany; b Division of Neurosurgery, Department of Biotechnology and Life Sciences, University of Brescia, Italy; c Cluster of Excellence: “Matters of Activity. Image Space Material”, Humboldt University, Berlin, Germany; d Berlin Institute of Health at Charité – University Hospital Berlin, BIH Biomedical Innovation Academy, Junior Digital Clinician Scientist Program, Berlin, Germany

**Keywords:** Concorde position, Supracerebellar infratentorial approach, Patient positioning, Pineal, Craniotomy, Posterior fossa surgery

## Abstract

**Introduction:**

The lateral supracerebellar infratentorial (SCIT) approach is an established corridor for lesions of the pineal region, posterior thalamus, and dorsolateral brainstem. However, the classic Concorde position may result in unfavorable surgeon ergonomics and less intuitive anatomical orientation during lateral SCIT procedures.

**Research question:**

Can a modified Concorde positioning technique improve surgeon ergonomics and provide a more natural anatomical orientation during lateral SCIT surgery while preserving the advantages of the classic Concorde position?

**Methods:**

This retrospective cohort study included patients who underwent the lateral SCIT approach in Antegrade Concorde position 04/2020-10/2024. The modified head fixation aligns the surgical trajectory of the lateral approach nearly vertically, allowing the surgeon to operate while standing at the level of the patient's pelvis. Surgical techniques and outcomes were systematically documented, and all perioperative complications were recorded.

**Results:**

Twenty-eight patients (40 ± 18 years) with cavernous malformations, metastases, pilocytic astrocytomas, and meningiomas being the most common pathologies were included. Lesions involved the thalamus, pineal region, pons, midbrain, and cerebellum and were all successfully accessed. Postoperative complications occurred in 13 patients (46%), mainly transient neurological deficits related to lesion location, including diplopia (25%), sensory disturbances (14%), and gait instability (11%). Three patients (11%) required external ventricular drainage. No positioning-related complications occurred. Mild bilateral pneumocephalus without clinical relevance was observed in 8 patients (29%).

**Discussion and conclusions:**

The Antegrade Concorde position improves ergonomics and feasibility of the lateral SCIT approach by restoring natural anatomical orientation and enabling a vertical surgical trajectory from a comfortable stance at the patient's pelvis.

## Highlights

• Novel head fixation enables lateral SCIT in the Concorde position.
• Allows surgeon positioning at buttock level instead of the vertex.
• Provides more natural anatomical orientation than classic Concorde setup.
• Facilitates comfortable and ergonomic microscopic lateral SCIT access.

## Abbreviations:

CN = : Cranial Nerve
CSF = : Cerebrospinal Fluid
CT = : Computed Tomography
MRI = : Magnetic Resonance Imaging
nTMS = : navigated Transcranial Magnetic Stimulation
SCA = : Superior Cerebellar Artery
SCIT = : Supracerebellar Infratentorial
VAE = : Venous Air Embolism

## 1 Introduction

First described by Horsley and Krause in the early 20th century, the supracerebellar infratentorial (SCIT) approach has evolved into a widely used workhorse technique for lesions of the pineal region and adjacent structures, including the pulvinar, mesencephalon, superior cerebellum, and vermis (Krause, 1926; Horsley et al.). In addition to tumors, it has been applied to vascular pathologies such as aneurysms, cavernous malformations, and arteriovenous malformations (de Oliveira et al., 2010; Oliveira et al., 2013; Page, 1977; Yasargil, 1984; Stein, 1971, 1979). However, the standard midline SCIT approach is limited by the high density of vermian bridging veins, the need to work around the vein of Galen, and the steep midline tentorial angle, which may necessitate venous sacrifice and increase the risk of cerebellar infarction or hemorrhage (Ammirati et al., 2002; Hernesniemi et al., 2008). This makes it particularly difficult to access paramedian targets (Mascitelli et al., 2018). To overcome these limitations, Yasargil proposed a paramedian and lateral variant of the SCIT approach in 1972 (Voigt and Yasargil, 1976). By following the natural downward slope of the cerebellar surface along the tentorium, the lateral approach reduces the surgical angle, minimizes cerebellar retraction, and involves fewer bridging veins (Ogata and Yonekawa, 1997). It facilitates safer access to lateral structures such as the ambient cistern, thalamus, parahippocampal region, tectal plate, and superior cerebellar peduncle (Choque-Velasquez et al., 2020).

Various patient positions have been described for midline and lateral SCIT approaches, with the semi-sitting position traditionally favored for its gravitational cerebellar relaxation, reduced venous pressure, and upright anatomical orientation (Choque-Velasquez et al., 2020; Tahta and Akalan, 2023). However, this position is associated with well-recognized risks, including venous air embolism (VAE), cerebrospinal fluid loss, and tension pneumocephalus, as well as ergonomic challenges related to extreme patient head flexion and elevated surgeon arm positioning (Sengupta et al., 2021; Saladino et al., 2017; Bithal et al., 2004; Buhre et al., 2000; Ture et al., 2018; Feigl et al., 2014). The Concorde position provides a simpler and safer alternative with a more streamlined setup and lower risk profile (Kobayashi et al., 1983). Nevertheless, in its classic form, the surgeon operates anterior to the vertex, resulting in an inverted anatomical view that complicates spatial orientation—particularly in paramedian SCIT approaches. Attempts to mitigate this by repositioning the surgeon laterally around the shoulder are ergonomically suboptimal and fail to restore a natural anatomical perspective (Kikuta et al., 2004; Kyoshima, 2002).

In this paper, we revisit the classic Concorde position and present a modified configuration that allows the surgeon to operate from a lateral stance beside the patient's pelvis. This repositioning promotes a more intuitive anatomical orientation, allows the surgeon to rest their forearms and enables parallel hand positioning, thereby enhancing ergonomic comfort and spatial awareness. To achieve that, the proposed Antegrade Concorde position incorporates adjusted head fixation that reorients the surgical trajectory for the paramedian SCIT into a near-vertical axis and renders the lateral infratentorial space horizontal from the surgeon's perspective. Notably, this approach retains the original Concorde position's key advantages—such as its rapid and straightforward setup—while effectively overcoming its primary spatial and ergonomic constraints.

## 2 Material and methods

### 2.1 Study design and patient population

This retrospective cohort study was approved by the ethics committee of Charité - University Medicine Berlin (No. EA2/228/24) and conducted in accordance with the Declaration of Helsinki. Owing to the retrospective study design, the requirement for informed patient consent was waived, except for the patient depicted in Fig. 4, from whom written informed consent for publication was obtained. Data collection and reporting followed the STROBE guidelines for observational studies (Von et al., 2007). All consecutive patients who underwent a lateral SCIT approach in the Antegrade Concorde position at the Department of Neurosurgery, Charité University Medicine Berlin, between 04/2020 and 10/2024 were identified. Demographic, clinical, and radiographic data were retrospectively extracted from medical records, including intraoperative complications and new postoperative neurological deficits. For preoperative planning in patients with brainstem pathologies navigated transcranial magnetic stimulation (nTMS)–based tractography was used to map relevant motor pathways at the brainstem level (Zdunczyk et al., 2020).

### 2.2 Surgical technique

**Fig. 1:**
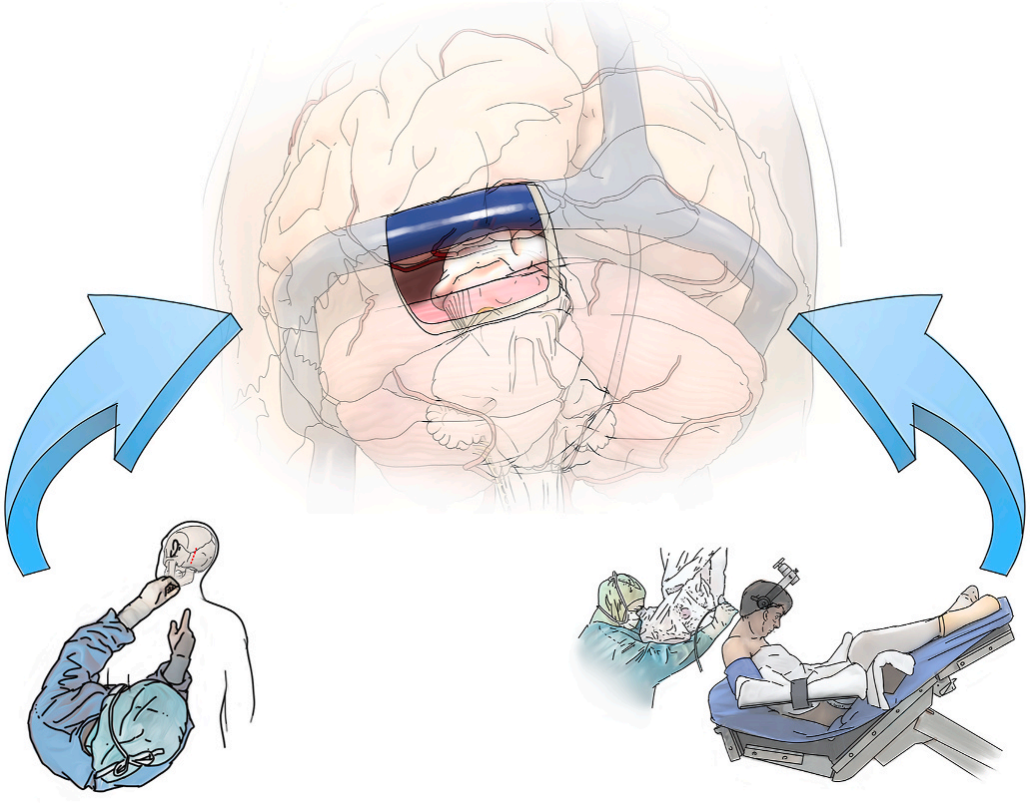
This figure demonstrates the concept of the Antegrade Concorde positioning (on the left), which allows the surgeon to achieve a natural, anatomically intuitive orientation toward the operative field. The specific modification of the head positioning enables the surgeon to work comfortably at the level of the patient's pelvis, aligning the visual and manual approach with the patient's anatomy. This orientation replicates the advantages of the traditional semi-sitting position (on the right) providing a familiar and ergonomic view of the surgical field — while retaining the simplicity, stability, and safety of the prone position.

**Fig. 2:**
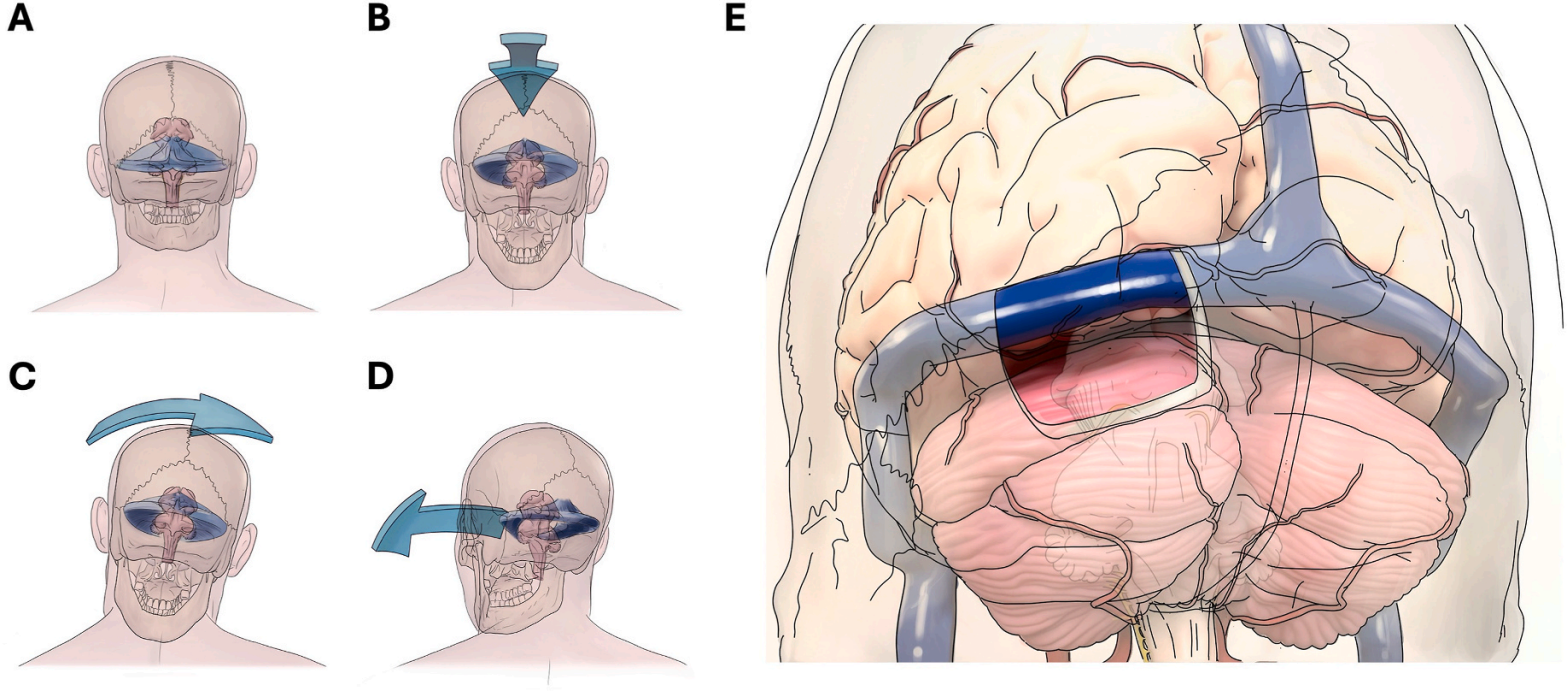
Demonstrates the key head positioning adjustments for integrating the lateral SCIT approach with the Antegrade Concorde position during a left-sided craniotomy, with the surgeon standing at the patient's left side near their buttocks. The neutral head position is shown in **A**. In **B**, the head is inclined to compensate for the steep angle of the tentorium. **C** demonstrates a sideways tilt toward the opposite side of the craniotomy, aligning the surgical plane so that the surgeon's hands work comfortably in a horizontal orientation. In **D**, the head is rotated contralaterally, shifting the craniotomy site away from the surgeon to enable a straight, vertical surgical trajectory when using the lateral SCIT approach. Finally, **E** shows the lateral craniotomy exposing the transverse sinus.

**Fig. 3:**
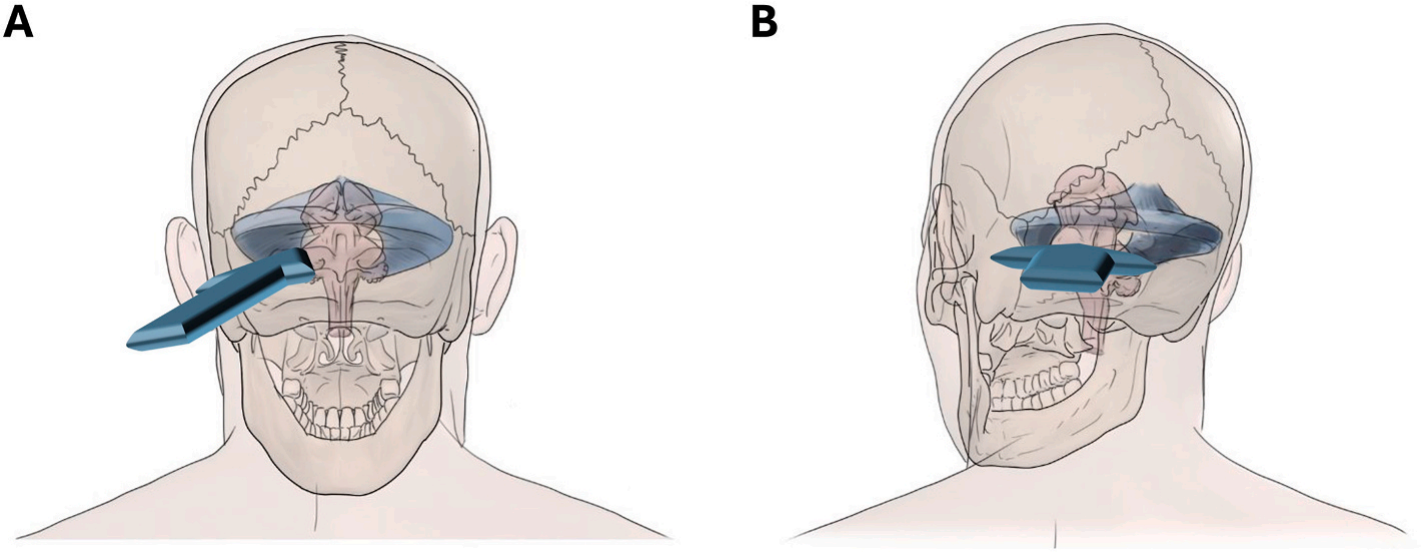
Illustrates the modification and alternative projection of the surgical trajectory. **A** shows the standard head fixation in the classic Concorde position, which involves neck flexion only. In this setup, the surgical trajectory is oblique, directed from lateral to medial. The surgeon is positioned at the vertex of the patient, and the trajectory is oriented toward the surgeon. This results in suboptimal ergonomics and poor visualization due to the inverted anatomical orientation. **B** presents the modified trajectory of the Antegrade Concorde position that is nearly perpendicular to the target. This is facilitated by a horizontal reorientation of the lateral infratentorial space. It allows the surgeon to operate in a natural anatomical alignment while standing beside the patient's pelvis, enabling parallel hand positioning and improved ergonomic comfort.

**Fig. 4:**
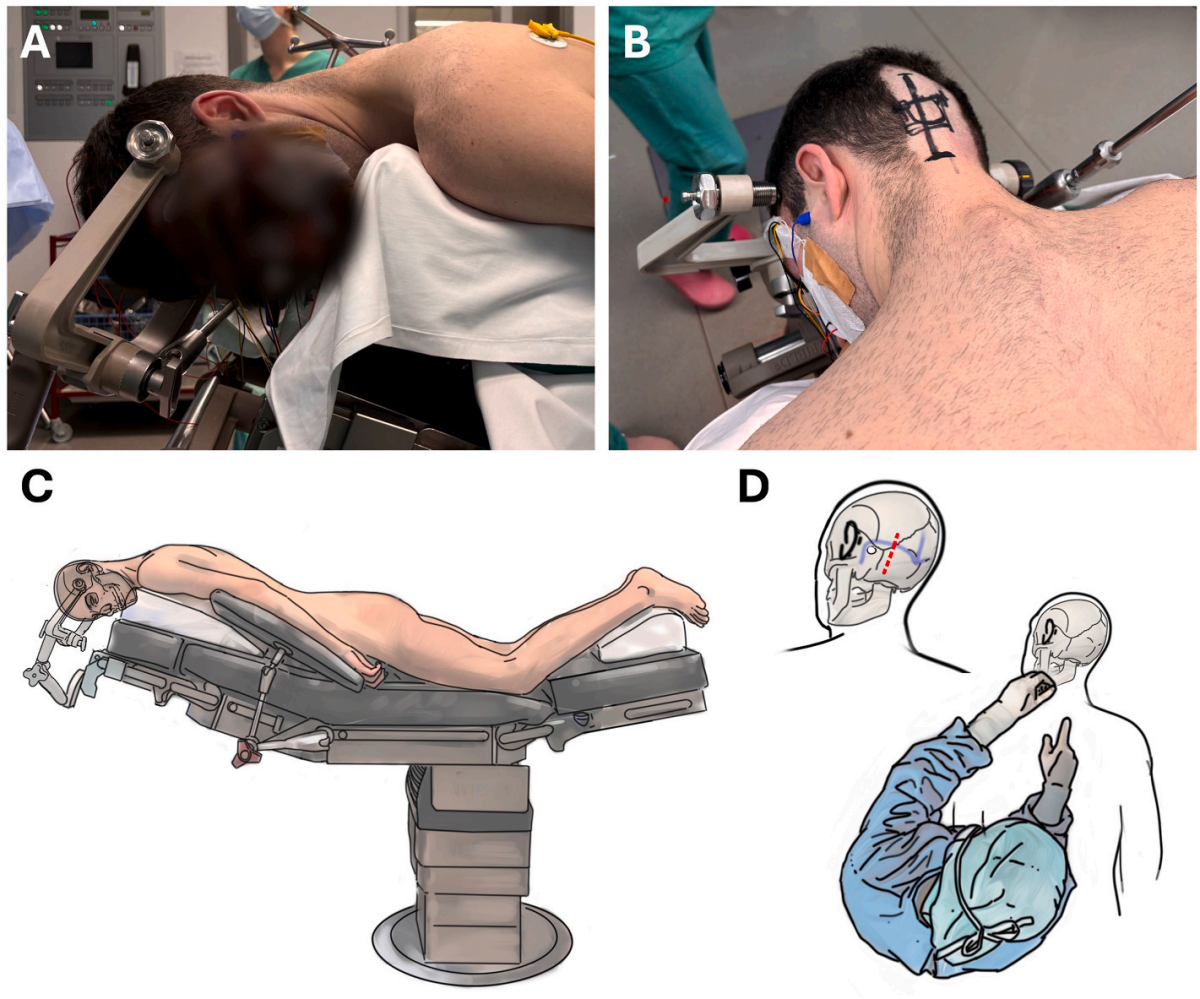
Presents the final patient positioning in the Antegrade Concorde position. **A** shows the adjusted head position in a lateral view, while **B** presents the surgeon's perspective. The planned lateral craniotomy is marked, with burr holes positioned along the transverse sinus on the inion-to-zygomatic-root line as surface landmarks. The planned incision is drawn laterally, directly over the intended craniotomy site. **C** depicts the final Antegrade Concorde position with the modified head adjustment, where the knees are flexed, and the upper body is elevated to achieve a reverse Trendelenburg position, ensuring the head is slightly above heart level. **D** provides a bird's-eye view of both the patient and the surgeon, highlighting how the modified head positioning and the surgeon's placement near the patient's buttocks allow for a comfortable working posture, enabling the surgeon to operate with their hands side by side without excessive leaning over the patient's back. The magnified view depicts the skin incision directly over the planned craniotomy site, located midway between the external occipital protuberance and the asterion.

The primary objective of this positioning concept is to modify the head fixation in such a way that the surgeon can operate while standing at the level of the patient's pelvis. This setup maintains a natural anatomic orientation, permits parallel hand positioning, and allows the surgeon to comfortably rest both forearms on the patient's shoulders, thereby optimizing ergonomics, precision, and operative control (Fig. 1). After induction of general anesthesia, the patient is placed prone with the body aligned along one edge of the operating table so that the torso extends fully to the lateral border. The ipsilateral arm is wrapped and secured against the body using drapes, eliminating obstruction and allowing the surgeon to stand near the patient's side. As in the standard Concorde position, the head slightly overhangs the cranial end of the table to ensure adequate submental clearance. The table is then tilted approximately 45° into a reverse Trendelenburg position while the knees are flexed. A strap around the thighs or buttocks prevents caudal slippage. The inclination can be adjusted so that the head remains above heart level to reduce venous congestion, while avoiding excessive elevation minimizing the risk of venous air embolism. This configuration also introduces a slight forward angulation of the surgical trajectory toward the surgeon, rather than a purely vertical orientation. As a result, the cerebellum descends gently away from the tentorium under gravity, reducing the need for retraction. All preparatory adjustments are completed before the head is rotated and tilted into its final operative orientation. For the lateral supracerebellar–infratentorial (SCIT) approach in the Antegrade Concorde position, the following modifications to head fixation are essential (Fig. 2, Fig. 3Fig. 4).

1. Inclination
  The head is inclined to compensate for the steep tentorial angle. This inclination, tailored to the patient's anatomy as determined on preoperative imaging, allows the surgical microscope to be positioned directly above the head so that the working axis remains vertical to the surgical field rather than oblique. This adjustment preserves a natural top-down trajectory and facilitates depth perception and bimanual coordination.
2. Lateral Tilting
  The head is then tilted laterally to neutralize the downward slope of the tentorium from medial to lateral. In combination with the surgeon's position at the level of the patient's pelvis, this maneuver brings the surgical plane into horizontal alignment, enabling both hands to operate symmetrically and in parallel. This reduces the need for awkward wrist flexion or crossing movements, thereby enhancing precision, stability, and ergonomic efficiency.
3. Contralateral Rotation
  Subsequently, the back of the head is rotated contralaterally so that the craniotomy site is directed slightly away from the surgeon. This orientation aligns the lateral supracerebellar–infratentorial (SCIT) corridor and the target region with the microscope axis, maintaining a straight and vertical surgical trajectory. This rotational adjustment is essential for performing the lateral SCIT approach in the Concorde configuration.
4. Surgeon's Position
  The surgeon stands adjacent to the patient's buttock, adopting a modified viewing angle that is nearly perpendicular to the space between the tentorium and the lateral cerebellar surface. This position—complementing the lateral head tilt—permits the surgeon to work with both hands side by side within the operative corridor, minimizing crossing maneuvers. The setup provides superior ergonomic balance, spatial orientation, and reduces fatigue during prolonged microsurgical dissection (Fig. 4).
  These modifications together enable the safe and efficient performance of the lateral SCIT approach in the Antegrade Concorde position, clearly distinguishing this positioning concept from previously described techniques.

Conversely to the conventional midline approach, the skin incision is made laterally, directly over the planned craniotomy site, midway between the external occipital protuberance and the asterion. Utilizing monopolar diathermy, dissection of the occipito-cervical muscles is performed longitudinally, and the semispinalis capitis muscle is detached from the superior nuchal line enabling broader retraction and wound exposure. Two burr holes are positioned directly on the transverse sinus. The lateral infratentorial craniotomy is then executed away from these burr holes. The transverse sinus is partially skeletonized to allow its visualization, which aids in preserving it and facilitates its mobilization and gentle cranial retraction after durotomy. The dural flap is then performed in a single curved fashion, based on the sinus. Retraction sutures along the tentorium might be used to enhance cranial retraction of the sinus to expand the surgical corridor.

Dynamic retraction with the suction device facilitates gentle exposure of the supracerebellar space, while sharp microsurgical dissection of arachnoid adhesions between the cerebellar and tentorial surfaces is performed until the quadrigeminal cistern is reached and opened. This step allows for CSF release, leading to relaxation and gradual downward mobilization of the cerebellar hemisphere under gravity. A lumbar drain may optionally be used to further assist cerebellar relaxation and mobility. With appropriate patient positioning and adequate CSF drainage from the basal cisterns, rigid retractors are unnecessary. The ergonomic advantage of the Antegrade Concorde position enables a retractorless technique, in which gentle dynamic retraction using the suction and bipolar instruments provides sufficient exposure. Following additional CSF egress from the pineal or quadrigeminal cisterns, cerebellar relaxation increases even further, facilitating safe dissection and minimizing traction-related injury.

## 3 Results

**Fig. 5:**
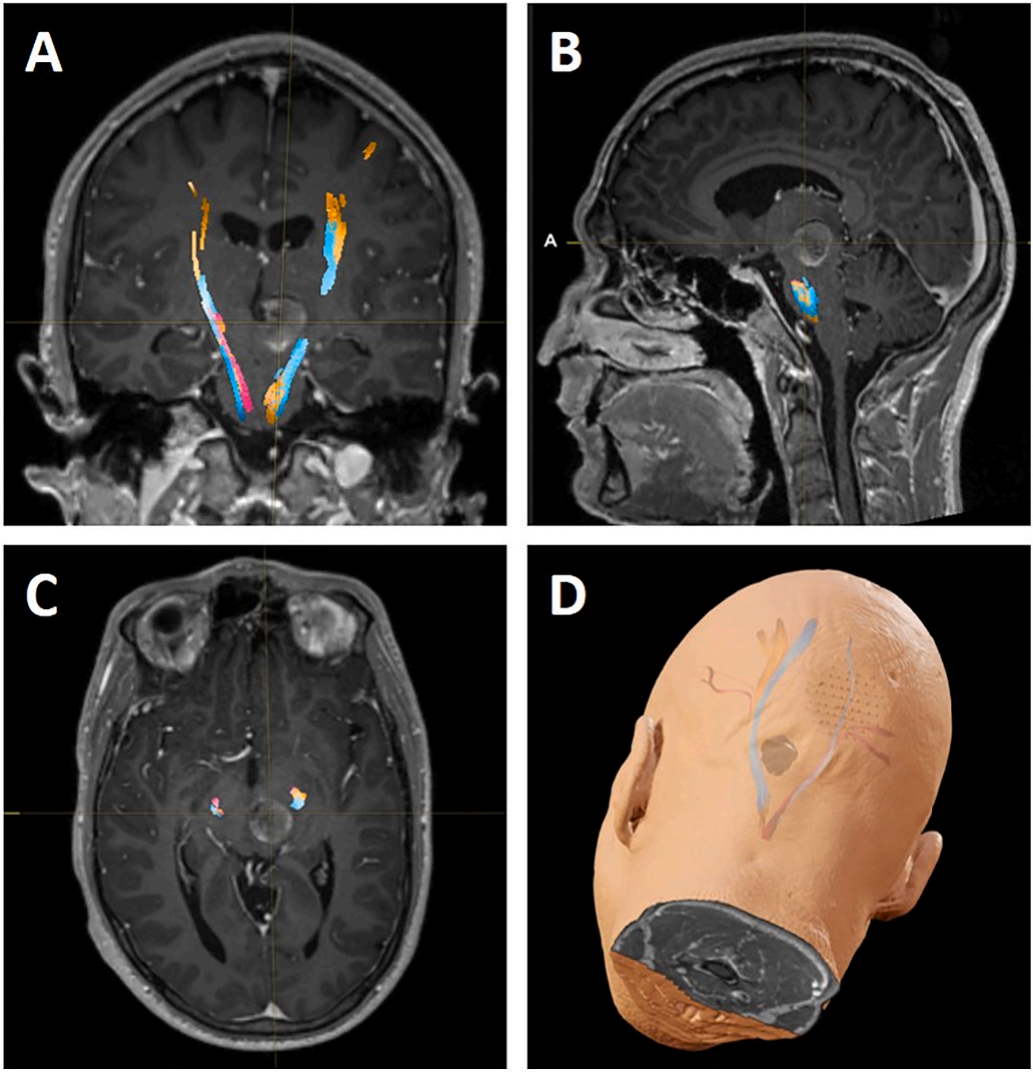
Presents an illustrative case of a 37-year-old patient with recurrent hemorrhages from a cavernous malformation in the left dorsal thalamus and mesencephalon. The patient exhibited progressive hemiparesis and decreased consciousness, necessitating surgical resection. To aid in surgical planning, navigated transcranial magnetic stimulation-based tractography was used to visualize the corticospinal tract, with blue representing motor pathways for the legs, pink for the face, and yellow for the hands. **A** shows the lesion in the coronal plane, **B** in the sagittal plane, and **C** in the axial plane. **D** provides an overview correlating with the surgeon's expected intraoperative view in the modified Concorde position.

**Fig. 6:**
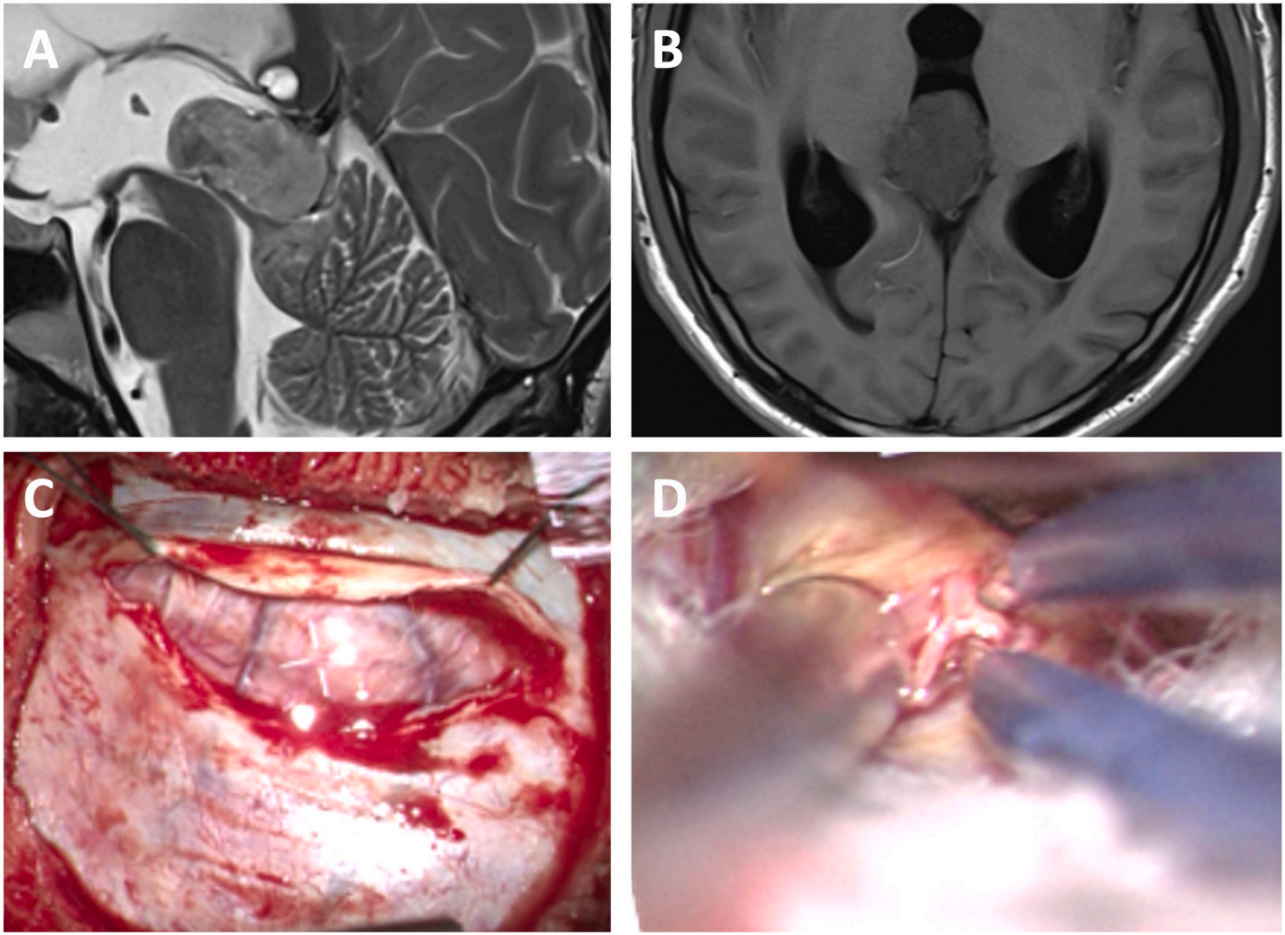
Presents a case of a 27-year-old patient with a pineoblastoma WHO Grade 4. The patient initially presented with severe headaches persisting for two weeks, along with nausea, mild anisocoria, and photophobia. **A** displays the findings in a T2 sequence in the sagittal plane, while **B** shows a contrast enhanced T1 sequence in the axial view. **C** illustrates the craniotomy of a right sided lateral SCIT approach, with the burr holes placed on the transverse sinus. The dura mater was carefully opened based towards the sinus and mobilized with retraction sutures. **D** depicts the view through the microscope, showing the initial stage of tumor removal. It is evident that the surgeon is able to perform precise, side-by-side hand movements with ease.

Between 04/2020 and 10/2024, 28 patients with a mean age of 40 ± 18 years and a female-to-male ratio of 1:1.6 were included. The cohort comprised patients with primary and secondary tumors of various entities and lesions were located in the thalamus, pineal region, pons, midbrain, and cerebellum, all of which were successfully accessed. Patient characteristics, including pathology and anatomical location, are summarized in Table 1. Two illustrative cases are presented in Fig. 5, Fig. 6.

**Table 1:** Overview of patient characteristics, with details on underlying pathology and anatomical location of the lesions.

Table 1. Patient characteristics	N = 28^a^
Age, years	40 ± 18
Sex	
Female	11 (39%)
Male	17 (61%)
**Pathology**	
Cavernous malformation	15 (54%)
Metastasis	3 (11%)
Pilocytic astrocytoma	2 (7%)
Meningioma	2 (7%)
Mesenchymal tumor	1 (4%)
Teratoma	1 (4%)
Pineal cyst	1 (4%)
Arteriovenous malformation	1 (4%)
Arteriovenous fistula	1 (4%)
Pineoblastoma	1 (4%)
**Localization**	
Pineal region	9 (31%)
Quadrigeminal plate	7 (24%)
Dorsal thalamus	4 (14%)
Pons	3 (10%)
Cerebellum	3 (10%)
Mesencephalon	1 (3%)
Roof fourth ventricle	1 (3%)

SD, standard deviation.

a Mean ± SD; n (%).

The mean skin-to-skin time was 111 ± 39 min. The mean perioperative preparation time, including patient positioning, head fixation, and sterile draping, was 50 ± 16 min. Preparation time progressively decreased throughout the series, from 55 ± 18 min in the first 18 procedures to 42 ± 9 min in the final 10 procedures. Surgeons reported no fatigue and were satisfied with the accessibility and ergonomic working posture. No patient required lumbar drain placement.

Several complications were documented, attributable either to the surgical approach itself or to the lesion's location. Bridging vein injury was reported in 6 patients (21%), with 2 cases of consecutive intracerebellar hemorrhage. Of the six documented bridging vein injuries, four occurred in patients with lesions located in the pineal or quadrigeminal region, corresponding to the midline supracerebellar corridor where bridging veins are most commonly encountered. In most cases, these events remained clinically uneventful and were not associated with additional neurological morbidity. One of the two patients with intracerebellar hemorrhage developed new-onset dysmetria, nystagmus, and progressive deterioration of consciousness, requiring temporary CSF diversion on postoperative day three, but made a full recovery over time. The other patient experienced new or worsened dysarthria, hemiparesis, hemi-hypesthesia, facial palsy, diplopia, and hypoacusis. These deficits were likely attributable, at least in part, to the eloquent mesencephalic location of the cavernous malformation rather than to the bridging vein injury alone. Only hemi-hypesthesia persisted at follow-up. Two patients without bridging vein injury exhibited delayed awakening and required temporary external ventricular drainage. Additionally, 13 patients (46%) experienced new or worsened early postoperative neurological deficits related to lesion location. In terms of patient positioning related complications, postoperative imaging revealed mild bilateral pneumocephalus in 8 patients (29%), representing incidental radiological findings. There was no midline shift or mass effect, confirming the absence of tension pneumocephalus. The radiological signs of pneumocephalus completely resolved in subsequent follow-up imaging. No additional complications related to patient positioning were observed. Table 2 provides a summary of complications associated with lesion location, surgical approach, and patient positioning.

**Table 2:** Perioperative complications.

Table 2. Perioperative complications	N (%)
**Related to lesion location**	
Diplopia	7 (25%)
Hypesthesia and/or dysesthesia	4 (14%)
Gait disturbance	3 (11%)
Vertical gaze palsy	2 (7%)
Dysmetria	2 (7%)
Hypoacusis	2 (7%)
Facial palsy	2 (7%)
Vertigo	1 (4%)
Dysarthria	1 (4%)
Nystagmus	1 (4%)
Anisocoria	1 (4%)
**Related to surgical approach**	
Bridging vein injury	6 (21%)
Cerebellar hemorrhage	2 (7%)
Requiring EVD	3 (11%)
**Related to patient position**	
Mild pneumocephalus	8 (29%)
Tension pneumocephalus	0 (0%)
Clinically manifest VAE	0 (0%)
Gravitational venous injury	0 (0%)

VAE, venous air embolism; EVD, external ventricular drain.

## 4 Discussion

The modifications to the classic Concorde position for the paramedian SCIT approach proposed in this study represent a fundamental conceptual advancement. By enabling the surgeon to stand laterally at the level of the patient's pelvis, this new configuration restores a natural and intuitive anatomical perspective. Through controlled flexion, tilting, and rotation of the head, the modified position effectively equalizes the angles within the lateral infratentorial corridor, thereby reorienting the surgical trajectory to an almost vertical axis. This alignment facilitates a more comfortable and stable working posture, enabling the surgeon to rest their arms and manipulate instruments in a parallel and ergonomically favorable manner. This adjustment addresses critical ergonomic and spatial challenges inherent to the traditional Concorde setup, in which the surgeon is positioned anteriorly at the vertex.

Patient positioning is crucial in neurosurgery, as it directly influences surgical access, trajectory alignment, and technical feasibility, while affecting patient safety and surgeon ergonomics during prolonged procedures (Balasa et al., 2020; Takasuna and Tanaka, 2015). The classic Concorde position represents the most widely adopted prone positioning technique for supracerebellar infratentorial approaches because it combines excellent midline exposure with the avoidance of the complications like VAE (Arthur et al., 2018). However, despite its procedural simplicity and favorable safety profile, the classic Concorde position has ergonomic limitations. The surgeon operates anterior to the vertex, resulting in an inverted anatomical view and an oblique surgical corridor dictated by the steep tentorial angle, often necessitating uncomfortable hand and arm positioning and impairing spatial orientation (Kikuta et al., 2004; Kyoshima, 2002). Furthermore, the classical Concorde position is primarily designed for midline trajectories and is less well suited for lateral or paramedian SCIT approaches, as the surgeon must work at an increasingly oblique angle to the surgical corridor, limiting instrument maneuverability and ergonomic comfort.

Several positions have been proposed for the lateral SCIT approach, including the park-bench, lateral decubitus, and semi-sitting positions (Awad et al., 2016). Among these, the semi-sitting position has traditionally been preferred because it facilitates gravitational cerebellar relaxation, cerebrospinal fluid drainage, and reduced venous bleeding (Oliveira et al., 2013). However, its use has declined in many centers due to the associated risks of complications such as VAE, hemodynamic instability, and tension pneumocephalus, as well as the substantial logistical demands and the need for specialized intraoperative monitoring with transesophageal echocardiography and a multiorifice central venous catheter (Sengupta et al., 2021; Saladino et al., 2017; Bithal et al., 2004; Buhre et al., 2000; Ture et al., 2018; Feigl et al., 2014). In addition, the steep tentorial angle necessitates an upward surgical trajectory with prolonged arm elevation, imposing considerable ergonomic strain on the surgeon and potentially compromising concentration and microsurgical precision during lengthy procedures (Ammirati et al., 2013).

The Concorde position may represent a safer alternative to the semi-sitting position by reducing position-related complications. Elevation of the head above heart level in reverse Trendelenburg decreases venous congestion without substantially increasing risk of VAE. In contrast, reported VAE rates are considerably higher in the semi-sitting position than in prone positioning, with values ranging from 41 to 56% versus approximately 11%, depending on pathology and surgical indication (Saladino et al., 2017; Konrad et al., 2022; Rushatamukayanunt et al., 2016; Tufegdzic et al., 2021). Although not all VAEs result in clinical sequelae, clinically relevant complications and hemodynamic instability requiring intraoperative repositioning have been reported in up to 7% of cases, which remains substantial for a risk solely attributable to patient positioning (Saladino et al., 2017; Himes et al., 2017).

Despite these advantages, the previously discussed limitations in ergonomics, surgical access, and anatomical orientation underscore that the classic Concorde position still offers room for further refinement. Historical advances, most notably Spetzler's modified park-bench position, have demonstrated the critical impact of optimized positioning on surgical exposure and outcomes (Baldwin et al., 1994; Lanzino et al., 2005). By combining head flexion, rotation, and tilting, Spetzler improved access for far-lateral approaches and illustrated how subtle adjustments can substantially enhance microsurgical feasibility. However, the park-bench configuration is associated with practical drawbacks, including extensive patient rotation, complex arm positioning, prolonged setup times, and potential impairment of venous return with increased risk of pressure-related complications and cervical strain. In this context, the Antegrade Concorde configuration represents a logical evolution, preserving the anatomical advantages of Spetzler's concept while offering greater procedural simplicity, safety, and surgeon comfort for posterior fossa and pineal region surgery.

The Antegrade Concorde position, which optimizes both head orientation and surgeon positioning, effectively addresses the ergonomic limitations of the classic Concorde setup. Unlike previous adaptations, such as those described by Takasuna et al., which adjusted the head position after the initial incision, our technique involves positioning the head in its final configuration before draping and incision (Takasuna and Tanaka, 2015). This approach not only simplifies the surgical setup but also enhances surgical orientation by allowing the surgeon to stand adjacent to the patient's pelvis. This way, the natural anatomical alignment is restored in an upright orientation, with an almost perpendicular surgical trajectory. This positioning facilitates a more ergonomic and intuitive visualization of the operative field, allowing the surgeon to comfortably support their forearms and minimizing the need for awkward hand postures or crossing movements.

Adequate gravitational cerebellar relaxation is achieved in the Antegrade Concorde position, as the tentorial slope is never fully vertical. Reverse Trendelenburg positioning with the head slightly above heart level places the surgical corridor at the highest point of the cranial vault, facilitating effective CSF drainage without the need for lumbar drainage (Sanai et al., 2010). After opening the basal cisterns, the cerebellum typically relaxes sufficiently to allow a wide and unobstructed operative corridor without fixed retractors, particularly along the cerebellomesencephalic fissure and ambient cistern where the natural medial-to-lateral slope further supports gravity-assisted exposure (de Oliveira et al., 2010). Although lumbar drainage may be considered as an adjunct in selected cases, it was not used in the present series. Adequate cerebellar relaxation was consistently achieved by optimized patient positioning and cerebrospinal fluid release from the basal cisterns, without the need for lumbar drainage or fixed cerebellar retractors. In contrast, lateral approaches in prone, park-bench, or lateral positions often rely on lumbar drainage to achieve adequate brain relaxation. While the semi-sitting position enables early cisternal opening and gravitational CSF outflow, it is associated with specific risks such as tension pneumocephalus, prolonged recovery, and increased stress on bridging veins (Ture et al., 2018; Feigl et al., 2014; King and Harmon, 1994). Postoperative intracranial air is likewise common after surgery in the semi-sitting position. In a large series of vestibular schwannoma procedures, Machetanz et al. identified postoperative pneumocephalus in almost all semi-sitting cases (Machetanz et al., 2020). Although tension pneumocephalus was uncommon, it occurred in 3.3% of patients and required air evacuation. Other series using the semi-sitting position have likewise reported a high incidence of postoperative pneumocephalus, although these postoperative air collections remained clinically asymptomatic and did not require surgical intervention (Al-Afif et al., 2024; Teping et al., 2021). In our series, mild postoperative pneumocephalus was observed in 29% of patients, whereas no patient developed tension pneumocephalus or required surgical intervention. All cases represented incidental radiological findings without mass effect, midline shift, delayed neurological deterioration.

Because the ergonomic benefits of the Antegrade Concorde position depend on the precise execution of these key modifications, an initial learning curve should be considered when adopting the technique. In our experience, successful implementation depends on careful attention to several positioning details, including true lateral patient positioning, slight overhang of the head and shoulders beyond the table edge, and precise adjustment of head inclination, rotation, and tilt to achieve the desired reorientation of the surgical axis. During the initial adoption of the technique, minor deviations from the intended positioning occasionally reduced its ergonomic benefits. Although the learning curve was not formally evaluated, reproducibility improved as the surgical team became more familiar with these technical aspects. Following an initial learning phase, preparation time stabilized at approximately 35–45 min in the later cases, suggesting increasing familiarity of the surgical and operating room team with the positioning technique. Therefore, standardized positioning protocols and dedicated training of the operating room team may facilitate consistent implementation and reduce inter-surgeon variability.

Compared with the technical complexity and perioperative risks of the semi-sitting position, this approach might offer a favorable balance between surgical exposure, safety, and procedural simplicity (Saladino et al., 2017; Ammirati et al., 2013). These findings emphasize the value of continuously optimizing established positioning concepts, integrating the advantages of the lateral SCIT approach with an ergonomically and hemodynamically safer prone configuration.

### 4.1 Limitations

The present study is limited by its retrospective single-center design and the relatively small, heterogeneous cohort comprising different pathologies and lesion locations. Consequently, postoperative neurological outcomes should be interpreted in the context of the underlying pathology, surgical complexity, and the surgical approach. Although postoperative imaging and clinical assessment frequently allowed anatomical correlation of neurological deficits, a clear distinction between lesion-related and approach-related morbidity was not always possible. Importantly, no major complications specifically attributable to the Antegrade Concorde positioning technique, such as clinically manifest VAE, were observed. Likewise, the proposed ergonomic advantages were not formally assessed using standardized ergonomic or surgeon-reported outcome measures but are based on the operative experience of the surgical team. Future prospective studies incorporating objective ergonomic assessments and performance metrics may further characterize the potential benefits of the Antegrade Concorde position.

## 5 Conclusions

The Antegrade Concorde position refines the classic Concorde setup by enabling the surgeon to operate from a lateralized stance at the level of the patient's pelvis. This orientation restores a more intuitive anatomical perspective with direct alignment along the surgical axis, allowing comfortable forearm support and parallel hand movement. Optimized head fixation redirects the lateral SCIT trajectory into an almost vertical working axis, which may facilitate depth perception, microsurgical maneuverability, and overall procedural control. By avoiding non-ergonomic arm positioning, complex setup, and patient rotation, this positioning strategy represents a feasible and ergonomic alternative for the lateral SCIT approach. Further prospective studies incorporating objective assessments of surgeon ergonomics, operative performance, and learning curve are warranted to validate these potential advantages.

This research did not receive any specific grant from funding agencies in the public, commercial, or not-for-profit sectors.

## Previous presentations

This work was presented at the 76th Annual Meeting of the German Society of Neurosurgery (DGNC), July 4, 2025, Hannover, Germany.

## Declaration on the use of artificial intelligence

ChatGPT (OpenAI, GPT-5.5) was used exclusively to improve the readability and language of the manuscript. The authors reviewed and edited the output and take full responsibility for the final content of the manuscript.

## Disclosure of funding

This research did not receive any specific grant from funding agencies in the public, commercial, or not-for-profit sectors.

## Declaration of competing interests

The authors declare that they have no competing interests.
